# The temporal foliar transcriptome of the perennial C_3_ desert plant *Rhazya stricta* in its natural environment

**DOI:** 10.1186/1471-2229-14-2

**Published:** 2014-01-04

**Authors:** Steven A Yates, Igor Chernukhin, Ruben Alvarez-Fernandez, Ulrike Bechtold, Mohammed Baeshen, Nabih Baeshen, Mohammad Z Mutwakil, Jamal Sabir, Tracy Lawson, Philip M Mullineaux

**Affiliations:** 1 School of Biological Sciences, University of Essex, Colchester CO4 3SQ, UK; 2 Department of Biological Sciences, Faculty of Science, King Abdulaziz University, P.O. Box 80203, Jeddah 21589, Kingdom of Saudi Arabia

**Keywords:** Next generation sequencing, Transcriptomics, Circadian clock, *Rhazya stricta*, Perennial desert plants, Heat stress, Salinity stress, C_3_ photosynthesis

## Abstract

**Background:**

The perennial species *Rhazya stricta* (*R. stricta*) grows in arid zones and carries out typical C_3_ photosynthesis under daily extremes of heat, light intensity and low humidity. In order to identify processes attributable to its adaptation to this harsh environment, we profiled the foliar transcriptome of apical and mature leaves harvested from the field at three time periods of the same day.

**Results:**

Next generation sequencing was used to reconstruct the transcriptome and quantify gene expression. 28018 full length transcript sequences were recovered and 45.4% were differentially expressed (DE) throughout the day. We compared our dataset with microarray experiments in *Arabidopsis thaliana* (Arabidopsis) and other desert species to identify trends in circadian and stress response profiles between species. 34% of the DE genes were homologous to Arabidopsis circadian-regulated genes. Independent of circadian control, significant overlaps with Arabidopsis genes were observed only with heat and salinity/high light stress-responsive genes. Also, groups of DE genes common to other desert plants species were identified. We identified protein families specific to *R. stricta* which were found to have diverged from their homologs in other species and which were over -expressed at midday.

**Conclusions:**

This study shows that temporal profiling is essential to assess the significance of genes apparently responsive to abiotic stress. This revealed that in *R. stricta*, the circadian clock is a major regulator of DE genes, even of those annotated as stress-responsive in other species. This may be an important feature of the adaptation of *R. stricta* to its extreme but predictable environment. However, the majority of DE genes were not circadian-regulated. Of these, some were common to other desert species and others were distinct to *R. stricta*, suggesting that they are important for the adaptation of such plants to arid environments.

## Background

The greater than 250,000 extant angiosperm species are a consequence of diversification that has driven adaptation to virtually all terrestrial ecosystems [1,2]. These include many with a wide range of challenging climatic zones compared with those in which we grow our major crop species. Challenging environments include those that have extremes of temperature, low and erratic precipitation and daily flooding with seawater [3,4]. Such zones have a low diversity of plant species as a consequence of the extensive adaptations required for successful growth and reproduction in such environments [5]–[8]. In these conditions, speciation is driven more by climate and other abiotic factors than by competition with other plant species [6,9,10].

Arid zones (60-250 mm of annual rainfall [11]), have a range of climatic and meteorological features which impact on the vegetation [12]. This ranges from rocky plateaux that permit the growth of only shallow rooted annuals and mesophyte ephemerals to dry river bed wadis that can support deep rooted perennials [12]–[14]. The study of perennial plants adapted to function for extended periods in arid environments could provide rewarding insights to help develop novel traits and genes for crop improvement in the face of abiotic factors such as heat, high light intensities, salinity and low nutrient and water availability [12]. Studies of such plants in their natural extreme environment are a contrast to the many laboratory-based experiments that focus on a single stress [3].

The advent of massively parallel DNA sequencing technologies and allied developments in bioinformatics has brought genome-scale studies of orphan plant species within the range of many laboratories [15]. Several plant species adapted to extreme environments have been subjected to such investigations. For example, genome sequencing of *Thellungiella parvula*, a species adapted to a saline and resource poor environment [16]; transcriptome analysis of the resurrection plant, *Craterostigma plantagineum*, for dessication tolerance [17]; a comparative survey of the transcriptome of two mangrove species, *Rhizophora mangle* and *Heritiera littoralis*[4] for multiple abiotic stresses and *Populus euphratica* a desert tree adapted to drought and salinity [18]. A feature of many of these species is that their adaptations are extensive, such as recovery from desiccation in resurrection plants and the morphological and biochemical developments associated with crassulacean acid metabolism (CAM) photosynthesis [17]–[19]. While such features are intrinsically worthy of study, such adaptations do not lend themselves readily to transfer to crop plants because the control of the underlying genes that determine these processes remain unknown.

In this study, we report an initial survey of the transcriptome of the perennial desert plant species *Rhazya stricta* Decne growing in its natural environment. *R. stricta* is a glabrous erect perennial evergreen shrub approximately 90 cm high with dense semi-erect branches [20] (see also Additional file 1). It is native to South Asia and the Middle East and belongs to the family Apocynacae (Asterid clade), which includes species of medicinal value [21,22]. *R. stricta* is common in arid zones at elevations of 100-700 m above sea level [14] and has been found to have improved growth in wadis [21], suggesting a deep root system, like other arid zone perennial species [12]. It is able to tolerate a wide range of soil mineral salt compositions by accumulating Na^+^, K^+^, Ca^2+^ and Cl^-^ in its leaves [21].

Our reason for studying the transcriptome of *R. stricta* is that it has a typical C_3_ photosynthetic physiology, which is able to function well under typical desert conditions of very high light intensities, temperatures and vapor pressure deficits [23,24]. The analysis of gene expression patterns may reveal mechanisms associated with stress tolerance and protection of the photosynthetic apparatus. This would identify adaptations that contribute to the success of *R. stricta* in its arid environment and may be worthy of future study and transfer to C_3_ crop species. This paper reports our initial survey and interpretation of changes in the patterns of global gene expression at three periods of the day and a comparison of these responses with published studies on transcriptome responses to stress in a range of plant species.

## Results

### Physiology

**Figure 1:**
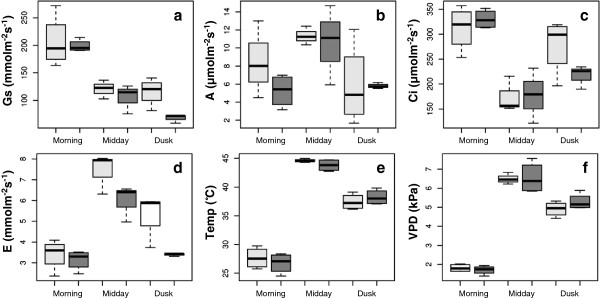
**Gas exchange measurements.** Mean instantaneous measurement of **(a)** stomatal conductance, **(b)** assimilation rate, **(c)** transpiration, **(d)** internal CO_2_ concentration **(e)** leaf temperature and **(f)** vapour pressure deficit captured on two leaves from 4 different plants. Measurements were taken on mature leaves (dark grey bars) and leaves from the apex (light grey bars) at three different times throughout the day.

Figure 1a-d illustrates the diurnal changes in photosynthesis assimilation rate (A), stomatal conductance (G_s_), internal CO_2_ concentration (*C*_i_) and transpiration rate (E) at three measurement periods: morning, midday and dusk. Also shown (Figure 1e-f) is the leaf temperature and vapour pressure deficit (VPD). G_s_ was greatest in the morning, and showed a midday depression which was maintained throughout the rest of the day (Figure 1a). A followed the diurnal pattern of irradiance (Figure 1b), with the highest values obtained at midday (despite reduced G_s_). C_i_ was greatly reduced during this period of high assimilation and low G_s_ suggesting that stomatal control of CO_2_ uptake limited A. It is noteworthy that E did not mirror G_S_ (Figure 1e and d). This was most likely due to the 18°C increase in leaf temperature and VPD increase to 6 kPa observed between morning and midday (Figure 1e).

### De novo transcriptome reconstruction and annotation

**Figure 2:**
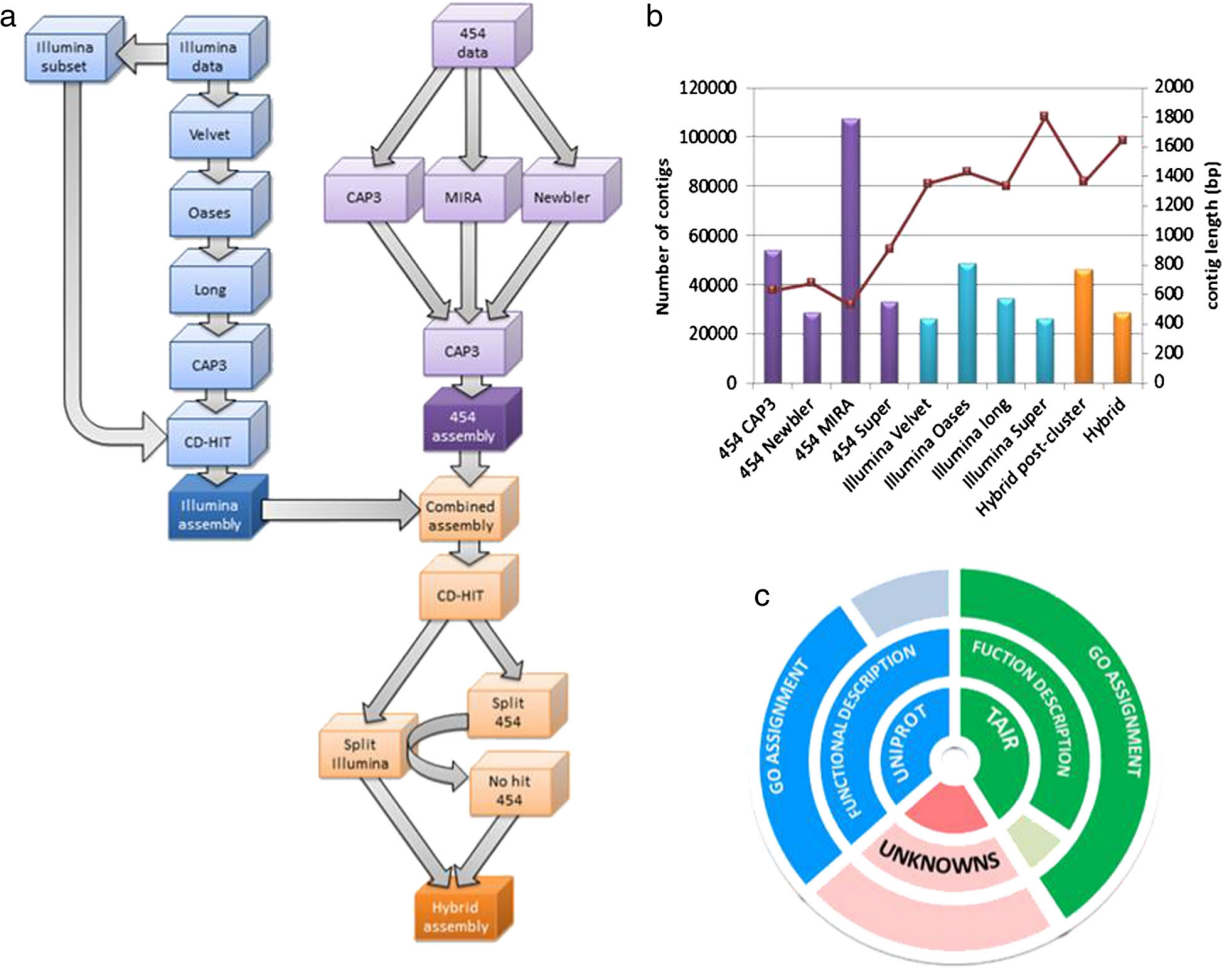
**Transcriptome reconstruction pipeline and results for assembly process at each stage and annotation metrics. a)** Flow diagram of de novo reconstruction using 454 pyrosequencing and Illumina data. Illumina data is shown in blue and 454 in purple, the combined data (hybrid assembly) is shown in orange. **b)** Summary statistics of each stage of de novo transcriptome reconstruction in *R.stricta*, uing 454 and Illumina sequencing data. Bars show the number of contigs created with the scale on the primary (left) axis and lines show mean contig length on the secondary (right) axis. The x-axis shows the assembly stages. Names pre-fixed with ‘454’ and ‘Illumina’ designate the source of the data for assembly (purple and blue respectively), ‘Hybrid’ means a combination of 454 and Illumina (orange). The remaining x-axis names show the software/stage used, except ‘Super’ which shows merging of the data. **C**, annotation metrics of *R.stricta* de novo transcriptome reconstruction, the layered pie chart shows the source of the annotion; TAIR, green; Uniprot, blue; and unannotated (unknowns) pink. The inner layer shows the source of transcripts annotated, the middle layer shows the proportion annotated with a functional description in solid colour and without in faded colour. The outer layer shows the proportion assigned at least one GO term in solid colour and without in faded colour. For unknowns the inner layer is solid colour to show unannotated and the middle and outer are by default faded as they contain no GO or description.

The *de novo* transcriptome assembly produced 28018 unique transcript sequences with a mean length of 1643 bp and N50 2199 bp (Figure 2b). 27617 sequences were uploaded to transcriptome shotgun assembly (TSA) Accession GAMW01000000, according to TSA criteria; the full list is available upon request. We were able to assign a functional description and gene ontology to 71.2% (19950) and 68.0% (19046) of the contigs, respectively (Figure 2c). The annotations are provided in Additional file 2 and the mean count per million (CPM) per leaf at each time in Additional file 3, and FDR significance results for 23037 quantifiable genes.

### RNA-seq

**Figure 3:**
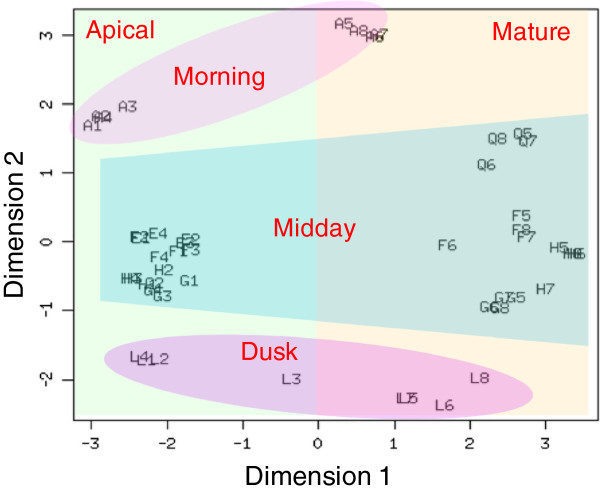
**Multi-dimensional scaling (MDS) plot of RNA-seq expression profiles in two dimensions.** For visual aid semi-transparent shapes have been overlaid to highlight the groupings of the data. On the right hand side all of the mature leaf samples are plotted (5-8) and on the left all of the apical leaves (1-4), coloured orange and green respectively. Then from top to bottom, the morning samples are at the top (A) (pink), the midday samples in the middle (E,Q,F,G,H) (Blue) and the dusk samples at the bottom (L) (pink).

To calculate expression, all reads were mapped against the *de novo* reconstructed transcriptome. To estimate the variation both between and within plants the biological coefficient of variation (BCV) was calculated (Table 1). The results show the BCV within plants was low (between 14-22%), but the BCV between plants was relatively greater. The latter is likely due to the inclusion of four plants, sampled over a four hour time frame. The next step was to use multivariate analysis to determine how well the gene expression profiles distinguished between the sampling time points and factors. This was done using a multi dimension scaling plot (MDS; Figure 3). The plot could clearly be divided into six groups which fitted the time and leaf type sampling factors. The number of quantifiable transcripts (see Methods) was 23037. Of these, 12761 genes were significantly differentially expressed in at least one pairwise comparison.

**Table 1:** Variation in RNA-seq data between and within plants, quantified as biological coefficient of variation (BCV)

**Factor**	**BCV**
A1-8	0.17
EQ1-8	0.2
F1-8	0.22
G1-8	0.14
H1-8	0.16
L1-8	0.21
All	0.19
Midday	0.34
Nested	0.29

Factors; A1-8, EQ1-8, F1-8, G1-8, H1-8, L1-8 shows BCV within plants when leaf type is taken as a factor. “All” is the same, but all plants and leaf types are considered as factors. For “Midday” this includes only midday samples (as defined in Methods) and uses leaf type as a factor. For “Nested”, all factors except plant, but leaf type is nested within time.

### Quantitative PCR validation

**Figure 4:**
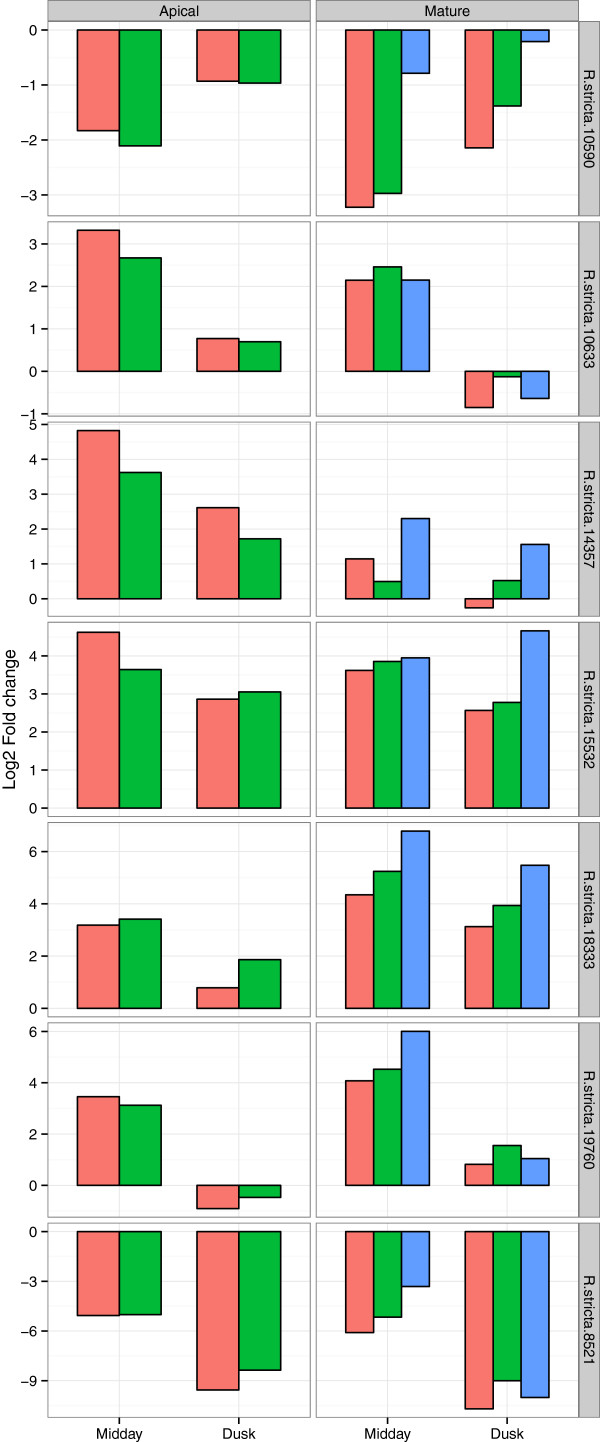
**Q-PCR validation, results show gene expression estimated by Q-PCR and RNA-seq.** Graphs show the log2 fold change in expression relative to morning, for midday and dusk samples. The Results are shown for QPCR estimates from the same samples in red and RNA-seq derived fold change shown in green. Also QPCR results shown from samples collect in the following year are shown in blue, where samples were only collected from mature leaves. The data is split between apical and mature leaves (left and right) and the seven genes tested.

Seven genes were validated using QPCR, the results were quantitatively similar to the RNA-seq data (Figure 4). Also the results were validated using mature leaf material collected from the following year (2012; Figure 4). The data from the 2012 samples were consistent between RNA-seq and previous QPCR estimates of gene expression from the 2011 samples.

### Gene ontology analysis

**Figure 5:**
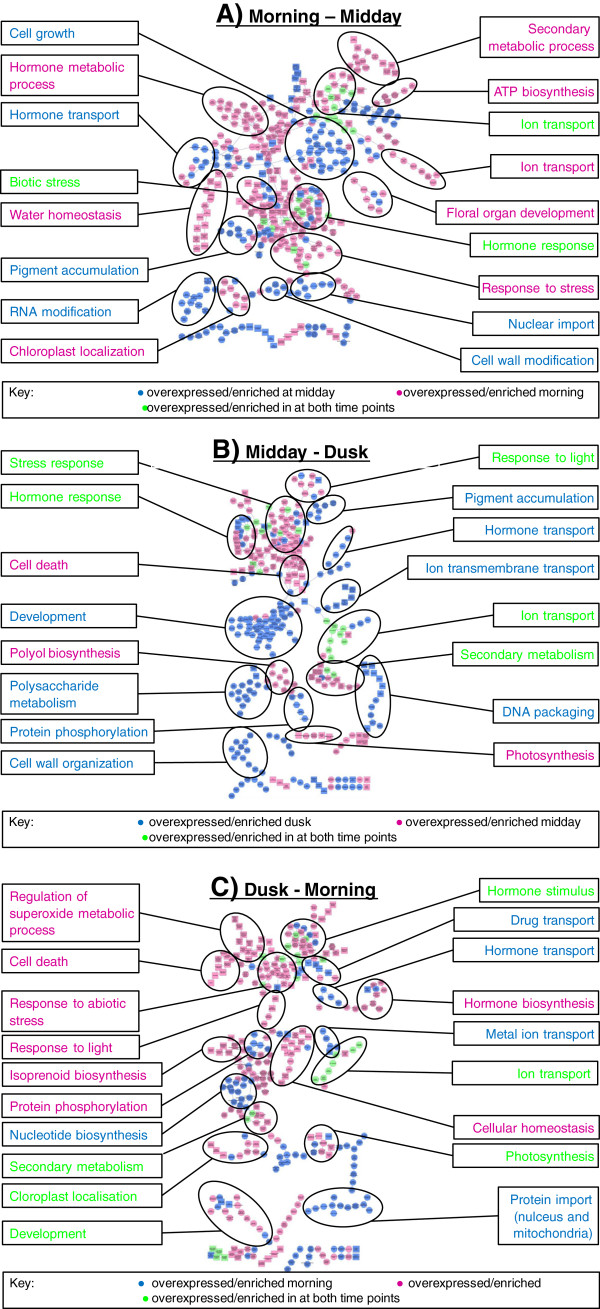
**Map of enriched GO terms at different times of the day, by pairwise comparison.** The map shows parent/child connections between GO terms. GO terms are coloured pink, blue and green depending on the enriched expression profile. The GO map is annotated with summary descriptions highlighted by larger circling of GO terms. The GO cluster descriptions colours correspond to the GO terms and which are described in the key. Pairwise comparisons are displayed in a, Morning – Midday; b, Midday – Dusk and c. Dusk – Morning

From the GO analysis (Figure 5) two trends were observed over the time course of the sampling. First, GO biological processes that increased throughout the day and then decreased from dusk to morning (Figure 5), included cell wall biogenesis (GO:0042546), pigment accumulation (GO:0043476), UV response (GO:0009411) and cell development (GO:0048468). In contrast response to stress (GO:0006950) and secondary metabolic process (GO:0019748) decreased morning to dusk. In the morning time point, GO terms relating to plastid localization (GO:0051644) and cellular homeostasis (GO:0019725) were over expressed (Figure 5c). As well as the trends described above, there were multiple signaling GO terms recruited throughout the day including protein phosphorylation, RNA modification (GO:0009451), DNA packaging (GO:0006323), hormone transport (GO:0009914) and ion transport (GO:0006810).

### Comparison with Arabidopsis microarray data

On a daily basis, *R. stricta* is exposed to a number of extreme abiotic challenges (see Introduction). The samples collected in this study represent a limited diel sampling which could include genes associated with responses to abiotic stress in other species. To test this proposition, a comparison was carried out of the *R. stricta* DE gene sets with microarray data from the response of the transcriptome of Arabidopsis subjected to several single and combined abiotic stresses [25]. For the comparisons of DE genes at the different sampling times and between apical and mature leaves, homologous genes between *R. stricta* and Arabidopsis were identified and the significance of the intersection of these datasets was calculated. The majority of abiotic stress comparisons were highly significant (Table 2). However, we reasoned instead that since *R. stricta* is adapted to these extreme conditions (see Introduction), then the apparent significance of this comparison might be misleading. Therefore, a comparison was made between *R. stricta* DE gene sets and those identified from a circadian regulated gene meta-analysis in Arabidopsis [26]. Assuming that *R. stricta* is adapted to its harsh habitat then many stress-associated genes could be differentially expressed because they were circadian regulated and not because they were responding to stressful environmental conditions. This is because circadian rhythm would not be disrupted in *R. stricta* leaves if it is acclimated to these extreme conditions. The results shown in Table 2, show approximately 29-34% of *R. stricta* DE genes are homologs to circadian regulated genes in Arabidopsis (based on homologous comparisons) and at all time points the comparisons were highly significant (Table 2; *P* < 0.01). When all circadian regulated genes were removed from the comparisons, the number of significant overlaps reduced, especially in apical leaves (Table 2). In mature leaves, overlaps only with heat, high light + salt and high light + heat Arabidopsis datasets were highly significant (*P* < 0.01). The genes identified with significant overlap are shown in Additional file 4. In the heat stress comparisons, midday to dusk DE genes encoding several small heat shock proteins and WRKY transcription factors (TFs) were evident. The morning to midday DE gene set overlapping with heat stress responsive genes included developmental homologs to *GIGANTEA* and *NO POLLEN GERMINATION RELATED1*. For the salt + high light from morning to midday a number of homologs to Arabidopsis genes encoding transporter proteins were found (AT5G12080, AT5G11800, AT5G57490, AT4G05120, AT5G15640). In the high light + heat, midday to dusk, four homologs to xyloglucan endotransglucosylase/hydrolases were found, which are involved in cell wall modification.

**Table 2:** **Comparison of *R. stricta *** **differentially expressed (DE) genes with Arabidopsis microarray datasets**

**Leaf type**		**Apical**			**Mature**		
**Treatment**	**DE homologs (Arabidopsis)**	**Morning to midday**	**Midday to dusk**	**Dusk to Morning**	**Morning to midday**	**Midday to dusk**	**Dusk to morning**
DE genes in *R. stricta*	*	5227	3223	5025	8301	4798	8116
DE homologs in *R. stricta*	*	3669	2142	3608	6163	3425	6185
Circadian	4611	1103^***^	743^***^	1210^***^	1803^***^	1133^***^	1906^***^
Heat	715	183^**^	130^***^	161^ns^	310^***^	182^***^	284^*^
Salt	293	83^*^	52^ns^	77^ns^	130^**^	61^ns^	119^ns^
Cold	464	136^***^	138^***^	146^***^	201^***^	139^***^	210^***^
Light	1049	274^***^	221^***^	283^***^	428^***^	296^***^	451^***^
Salt + Light	1153	269^ns^	208^***^	263^ns^	523^***^	282^***^	476^***^
Cold + Light	833	217^***^	175^***^	236^***^	333^**^	241^***^	373^***^
Heat + Light	782	196^*^	148^***^	165^ns^	348^***^	202^***^	291^ns^
Heat – circadian	504	126^*^	82^***^	99^ns^	217^***^	112^*^	185^ns^
Salt – circadian	209	56^ns^	31^ns^	47^ns^	89^*^	33^ns^	79^ns^
Cold – circadian	288	73^ns^	62^***^	70^ns^	115^ns^	62^ns^	107^ns^
Light – circadian	635	140^ns^	83^ns^	116^ns^	240^*^	129^ns^	229^ns^
Salt + Light – circadian	779	187^**^	125^***^	153^ns^	357^***^	170^**^	300^***^
Cold + Light – circadian	480	108^ns^	66^ns^	91^ns^	176^ns^	101^ns^	176^ns^
Heat + Light – circadian	543	119^ns^	90^***^	97^ns^	215^**^	121^*^	180^ns^

Circadian genes refer to meta-analysis of circadian regulated genes (Covington *et al.,* 2008). The table shows the Arabidopsis treatment (with or without circadian genes) against two leaf types apical and mature leaves and the pairwise comparisons between time points (morning, midday and dusk). The results show number of homologs and results of hypergeometric test, which is Bonferroni corrected for multiple testing p-values; significance is shown by * < 0.05, ** < 0.01, *** < 0.001 and ns > 0.05. The top row shows number of *R. stricta* genes DE and the remainder of the table shows numbers of homologous genes between *R. stricta* and Arabidopsis*.*

### Hormone synthesis

**Figure 6:**
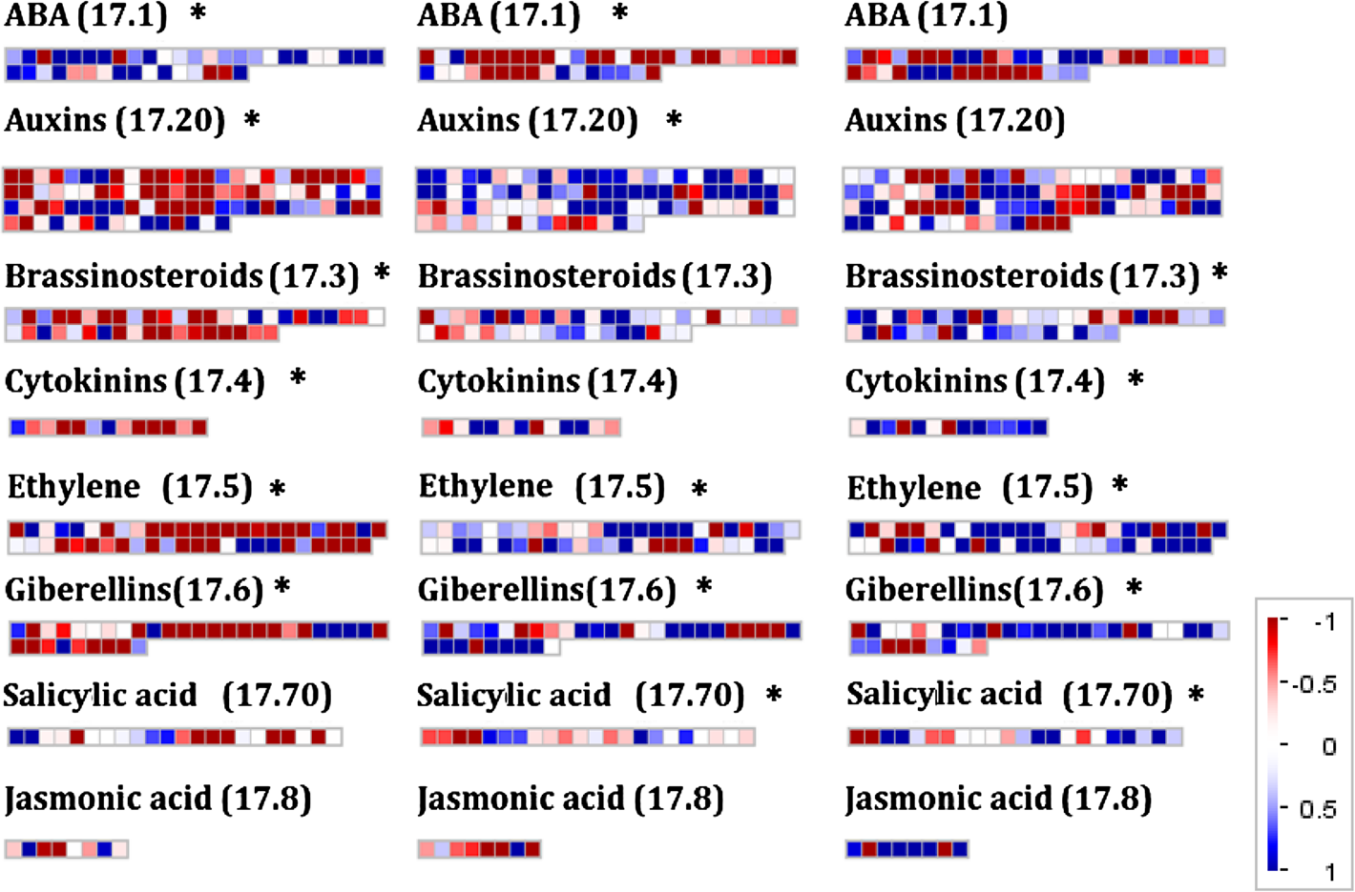
**Mapman diagrams for pairwise comparisons of hormone synthesis gene expression data.** (Log(2)FC) at different time points (from – to). Diagram shows comparisons in mature leaves, where blue is increased expression and red is decreased. The data is divided between Mapman Bin codes corresponding hormone synthesis pathway genes, which were identified as being enriched from Mapman Wilcoxon rank sum test. The plots show the hormone synthesis group and the relevant Bin code (17.×) and * to indicated significant enrichment or not. Each square represent s a single gene.

To investigate enzymes of hormone metabolism, the program Mapman was used and the results for mature leaves are shown in Figure 6, from which, three trends in the data were observed. First, the expression of genes encoding abscisic acid (ABA) biosynthesis enzymes increased from morning to midday, and then fell. The abundance of transcripts coding for NCED and ZEP peaked at midday. NCED and ZEP catalyse important reactions in the ABA biosynthetic pathway [27,28]. In contrast, most other DE genes encoding hormone metabolism decreased their abundance from morning to midday and then increased over the day. A closer inspection of the expression of DE genes encoding enzymes of hormone biosynthesis was conducted (Table 3). Transcripts encoding two isoforms of ACC synthase and one ACC oxidase respectively, which are determinants of ethylene synthesis [29] were the inverse of the expression pattern of ABA synthesis genes (Table 3). Transcripts coding for AOS, AOC and OPR3, key enzymes of jasmonic acid (JA) biosynthesis [30] were found. Both AOS and AOC transcripts showed a decrease in abundance from morning through the day, and OPR3 transcript abundance rose over the same period. The isochorismate pathway synthesises salicylic acid in chloroplasts [31]. Annotation revealed two genes encoding ICS genes whose transcript abundance increased from morning to dusk and thus differed in their pattern of expression compared with other hormone biosynthesis genes (Table 3).

**Table 3:** **Differentially expressed genes in *R. stricta *** **discussed in the Results section**

**ID**	**Group**	**Abv**	**Description**	**LogFC Apical Morning – Midday**	**LogFC Apical Midday - Dusk**	**LogFC Apical Dusk – Morning**	**LogFC Mature Morning - Midday**	**LogFC Mature Midday - Dusk**	**LogFC Mature Dusk - Morning**
17395	HS	ACC oxidase 1	1-aminocyclopropane-1-carboxylate oxidase	-1.92^***^	1.02^***^	0.89^*^	-0.97^***^	-0.51^ns^	1.48^***^
4650	HS	ACC oxidase 2	1-aminocyclopropane-1-carboxylate oxidase	-2.72^***^	2.14^***^	0.58^ns^	-3.82^***^	1.05^**^	2.77^***^
11855	HS	ACC1	1-aminocyclopropane-1-carboxylate synthase	-0.58^**^	-0.29^ns^	0.87^**^	-1.27^***^	-0.22^ns^	1.48^***^
7628	HS	ACC2	1-aminocyclopropane-1-carboxylate synthase	-0.33^ns^	-0.35^ns^	0.69^**^	-0.39^*^	-0.61^***^	1.01^***^
22227	HS	AOC	Allene oxide cyclase	-0.81^***^	0.94^***^	-0.13^ns^	-1.21^***^	1.31^***^	-0.09^ns^
2272	HS	AOS	Allene oxide synthase	-0.69^ns^	-1.18^ns^	1.87^*^	-2.21^***^	-0.38^ns^	2.59^***^
11383	HS	ICS1	Isochorismate synthase	0.76^ns^	0.07^ns^	-0.83^ns^	0.93^*^	0.41^ns^	-1.35^**^
13155	HS	ICS2	Isochorismate synthase	0.71^ns^	0.03^ns^	-0.74^ns^	0.87^*^	0.39^ns^	-1.26^**^
11166	HS	IPT2	Isopentenyltransferase	0.90	-0.21^ns^	-0.70^**^	0.48^*^	-0.31^ns^	-0.16^ns^
12704	HS	NCED	9-cis-epoxycarotenoid dioxygenase	1.26	-2.56^***^	1.30^ns^	0.56^ns^	-3.39^***^	2.83^***^
16795	HS	OPR3	12-oxophytodienoate reductase	1.38	-0.54^ns^	-0.83^ns^	-0.03^ns^	0.07^ns^	-0.04^ns^
10633	HS	ZEP	Zeaxanthin epoxidase	2.62	-2.13^***^	-0.50^ns^	2.26^***^	-2.75^***^	0.50^ns^
14357	HS	IPT	Isopentenyltransferase	3.55	-2.14^***^	-1.41^*^	0.76^ns^	-0.52^ns^	-0.25^ns^
8521	TF	CCA1	circadian clock associated1	-4.78	-3.80^***^	8.58^***^	-3.94^***^	-5.43^***^	9.37^***^
15403	TF	CRF2a	cytokinin response factor 2	-0.14	-0.45^ns^	0.59^ns^	1.94^***^	0.11^ns^	-2.05^***^
15603	TF	CRF2b	cytokinin response factor 2	-0.87^**^	0.84^**^	0.02^ns^	-2.48^***^	0.11^ns^	2.37^***^
15532	TF	DREB2	dehydration response element binding factor 2	3.66^***^	-0.80^ns^	-2.86^***^	3.71^***^	-1.44^***^	-2.27^***^
10837	TF	PRR5a	pseudo response regulators 5	1.90^***^	-1.41^***^	-0.49^ns^	1.73^***^	-0.88^***^	-0.85^**^
9003	TF	PRR5b	pseudo response regulators 5	1.87^***^	-1.39^***^	-0.49^ns^	1.70^***^	-0.87^**^	-0.83^**^
11103	TF	PRR7a	pseudo response regulators 7	3.48^***^	-1.05^***^	-2.43^***^	2.89^***^	-0.72^*^	-2.18^***^
7261	TF	PRR7b	pseudo response regulators 7	1.87^***^	-1.32^***^	-0.54^ns^	1.85^***^	-1.83^***^	-0.01^ns^
10590	TF	RAP2.12a	RAP2.12	-2.18^***^	0.99^***^	1.19^***^	-3.20^***^	1.45^***^	1.75^***^
16151	TF	RAP2.12b	RAP2.12	-2.18^***^	1.00^***^	1.18^***^	-3.24^***^	1.50^***^	1.74^***^
13680	TF	RAP2.4a	RAP2.4	-0.56^*^	-0.67^*^	1.23^***^	-1.38^***^	-0.42^ns^	1.79^***^
6139	TF	RAP2.4b	RAP2.4	-1.25^***^	0.61^*^	0.64^ns^	-1.76^***^	-0.40^ns^	2.17^***^
7222	TF	TOC1b	timing of CAB expression1	0.33^ns^	0.08^ns^	-0.41^ns^	0.07^ns^	0.40^*^	-0.46^ns^

Showing *R. stricta* contig identifier in first column, a code for section of results discussed (HS, hormone synthesis; TF, transcription factor) in second column, abbreviation (Abv) and description in third and fourth columns respectively. The remaining columns show the log(2) fold change between time points (from – to) within leaf type, along with FDR corrected significance test (<0.05 = *, <0.01 = **, <0.001 = ***, >0.05 ns).

### Transcription factors

**Figure 7:**
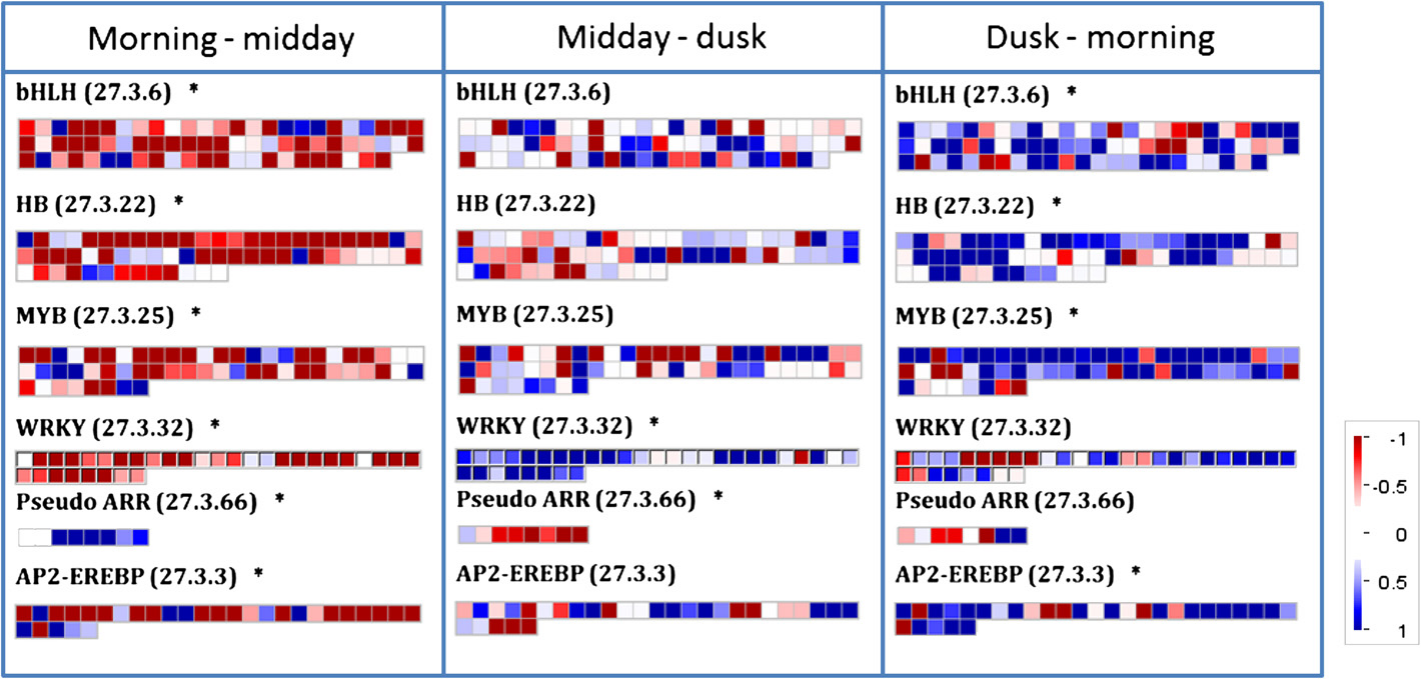
**Mapman diagrams for pairwise comparisons of transcription factor gene expression data.** (Log(2)FC) at different time points (from – to). Diagram shows comparisons in mature leaves, where blue is increased expression and red is decreased. The data is divided between Mapman Bin codes corresponding transcription factor family genes, which were identified as being enriched from Mapman Wilcoxon rank sum test. The plots show the hormone synthesis group and the relevant Bin code (17.×) and * to indicated significant enrichment or not. Each square represents a single gene.

Mapman analysis was again used to investigate TF gene families. This was done by identification of Wilcoxon rank sum test enriched Bin codes. The results are shown in Figure 7 for six significant families (as annotated by Mapman). The results were divided into three groups based on differential expression patterns of their genes. The basic helix loop helix (bHLH), homeobox domain (HB) and MYB TFs and AP2-EREBP gene families were found to significantly decrease in expression from morning to midday (Table 3). These large families are involved in a wide range of processes such as developmental and stress responses [32]–[34]. The second group is the WRKY TF gene family members which are typically associated with responses to biotic stress but also to abiotic stress [35]–[37]. This TF gene family showed a very strong decrease from morning and then a strong increase of all members between midday and dusk (Table 3). Third, the Arabidopsis response regulators (ARR) TF gene family [38] showed the opposite expression profile to the WRKY TF gene family, increasing expression from morning to midday and then a strong decrease from midday to dusk (Table 3).

The Apelta2 (AP2) TF family is large with many sub-groups (Riechmann & Meyerowitz, 1998) which were represented among the DE genes (Table 3). Two genes encoding homologs of RAP2.12 were found (*RAP2.12a & RAP2.12b*; Table 3) which is an oxygen sensing TF [39]. The expression of both genes decreased during the day (Table 3). Similarly genes encoding two homologs of RAP2.4 were found (*RAP2.4a & RAP2.4b*) and their transcript abundance decreased from morning to dusk (Table 3). Both genes in Arabidopsis are down-regulated by high light [40]. Genes for members of the cytokinin response factor (CRF) TF families were found to be differentially expressed. Two CRF2 homologs (*CRF2a* and *CRF2b*) decreased at midday. Like the gene coding for CRF2, a DREB2 gene homolog was found to increase expression over midday. This class of DREB is associated with osmotic stress [41].

The group annotated as genes encoding “pseudo” ARRs by Mapman was expanded to look at other circadian clock TF genes. Two homologs coding for PRR5 and PRR7 (*PRR5a*, *PRR5b* and *PRR7b*) were found (Table 3) and all showed a similar trend in differential expression, with peak expression at midday. This was shown for *PRR5* in Arabidopsis although *PRR7* showed peak expression in the morning [42]. The expression of two other well-characterised circadian clock genes were also investigated *TOC1* (two homologs in *R. stricta TOC1a* and *TOC1b*) and *CCA1*. The expression profile of these two genes is the same as that found in Arabidopsis (Table 3) [43].

### Overlap with transcriptomic datasets from *P. euphratica* and *C. plantagineum*

Comparison of DE genes from *P. euphratica*[18] and *C. plantagineum* showed highly significant similarity (*P* < 0.001; Hypergeometric Test) with those from *R. stricta* (Table 4). Prominent groups of genes uncovered in common with all three datasets were those encoding cysteine proteases, RuBisCo activase, chlorophyll AB binding proteins and galactinol synthase isoforms. Cysteine proteases are expressed in response to abiotic and biotic stress and during senescence [44]. RuBisCo activase has been found to release inhibitory sugar phosphates from RuBisCo and acts as a chaperone with thylakoid-bound ribosomes under heat stress [45]. Galactinol synthase, an enzyme of raffinose biosynthesis is involved in protection against heat stress [46,47]. Specifically to DE genes in common with the *P. euphratica* dataset (Table 4), a plastid terminal oxidase (PTOX) gene was found to be differentially expressed. PTOX is involved in electron transfer from linear photosynthetic electron transport to O_2_ and thus may contribute to the dissipation of excitation energy and protection against high light and heat stress [48,49].

**Table 4:** **Comparison with *P. euphratica *** **dataset and pairwise comparisons in *R. stricta***

**Genbank ID**	**Function**	**Apical leaves**			**Mature leaves**		
		**Morning – midday**	**Midday – dusk**	**Dusk - morning**	**Morning – midday**	**Midday – dusk**	**Dusk - morning**
		15 ^*^	16 ^***^	22 ^***^	25^*^	20 ^***^	26 ^***^
AJ780423	Cysteine protease	0	0	0	1	1	0
AJ780552	Cysteine protease	0	0	0	1	1	0
AJ780577	Cysteine protease	0	0	0	1	1	0
AJ771356	Sporulation protein-related	0	0	1	1	1	1
AJ771208	Polyubiquitin-like protein	1	0	0	1	0	0
AJ780294	Cysteine protease	0	0	1	0	0	0
AJ779452	Cysteine protease	0	0	1	0	0	0
AJ778920	Putative beta-1,4-N-acetylglucosaminyltransferase	0	0	0	1	0	1
AJ770693	Metallothionein type 3b	0	1	1	0	0	0
AJ772333	ATP-dependent Clp protease ATP-binding subunit clpA	0	0	0	1	0	0
AJ779386	Osmotin-like protein	0	0	0	1	0	1
AJ779694	Plastid terminal oxidase	1	0	1	1	0	0
AJ778685	Aluminium induced protein	0	1	0	0	1	1
AJ767463	Putative phospholipase C	1	0	1	0	1	1
AJ778911	Alanine aminotransferase	0	0	0	1	0	1
AJ769778	Beta-amylase	1	1	1	1	1	1
AJ769912	Granule-bound starch synthase	0	1	1	0	1	1
AJ769227	Galactinol synthase, isoform GolS	0	0	0	0	1	1
AJ767459	Galactinol synthase, isoform GolS	0	0	0	0	1	1
AJ771722	Transketolase, chloroplast	0	0	1	0	0	1
AJ770033	Ferritin	1	0	1	1	1	1
AJ771629	Flavonol 3-O-glucosyltransferase	1	1	1	1	1	1
AJ780435	Aldehyde dehydrogenase	0	0	0	0	0	0
AJ776096	Alcohol dehydrogenase	1	1	0	1	0	0
AJ768966	Glutamine synthetase	0	0	0	1	0	1
AJ767241	Ribulose bisphosphate carboxylase/oxygenase activase	1	1	1	1	1	1
AJ780215	Endomembrane-associated protein	0	1	1	0	0	0
AJ773744	1,4-Benzoquinone reductase-like, Trp repressor binding protein-like	0	0	0	1	1	1
AJ780732	Drought responsive ATP-binding motif containing protein	1	1	1	1	1	1
AJ778477	High mobility group B3 protein	1	1	0	1	1	1
AJ777667	Chalcone synthase	1	1	1	0	0	1
AJ776763	Chalcone synthase	1	1	1	0	0	1
AJ768632	Lipid transfer protein	0	0	1	0	0	1
AJ772852	Proline-rich protein/Lipid transfer protein	1	0	1	1	0	1
AJ771577	Putative aquaporin (tonoplast intrinsic protein gamma)	0	1	1	1	1	1
AJ774243	Trans-cinnamate 4-monooxygenase	1	1	1	1	1	1
AJ770887	Chlorophyll a/b-binding protein	1	1	1	1	1	1
AJ775526	Glutathione S-transferase	0	1	1	0	1	1
AJ775425	Proline-rich protein	0	0	0	1	0	0
AJ773383	Proline-rich protein	0	0	0	1	0	0

Table shows *P. euphratica* Genbank codes and functional description (columns 1-2 respectively), columns 3-5 show overlap between apical leaves and columns 6-8 for mature leaves. The row below indicates the pairwise comparison where significant DE genes were found. Below shows the number of homologs detected and hypergeometric test for overlap. The main body of the table indicates if at least one DE homolog was detected by ‘1’ and no homologous DE genes by ‘0’.

### Orthologous and novel proteins

**Figure 8:**
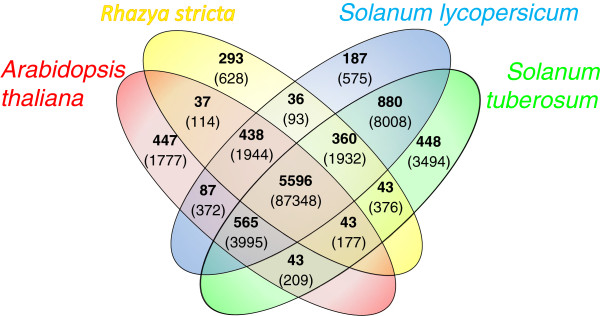
**Clusters of orthologous proteins between *A.thaliana*, *R.stricta*, *S.lycopersicum *and *S. tuberosum*, using OrthoMCL; shown in red, yellow, blue and green, respectively.** Within the Venn diagram; bold shows the number of orthologous groups and within brackets shows the number of proteins.

**Figure 9:**
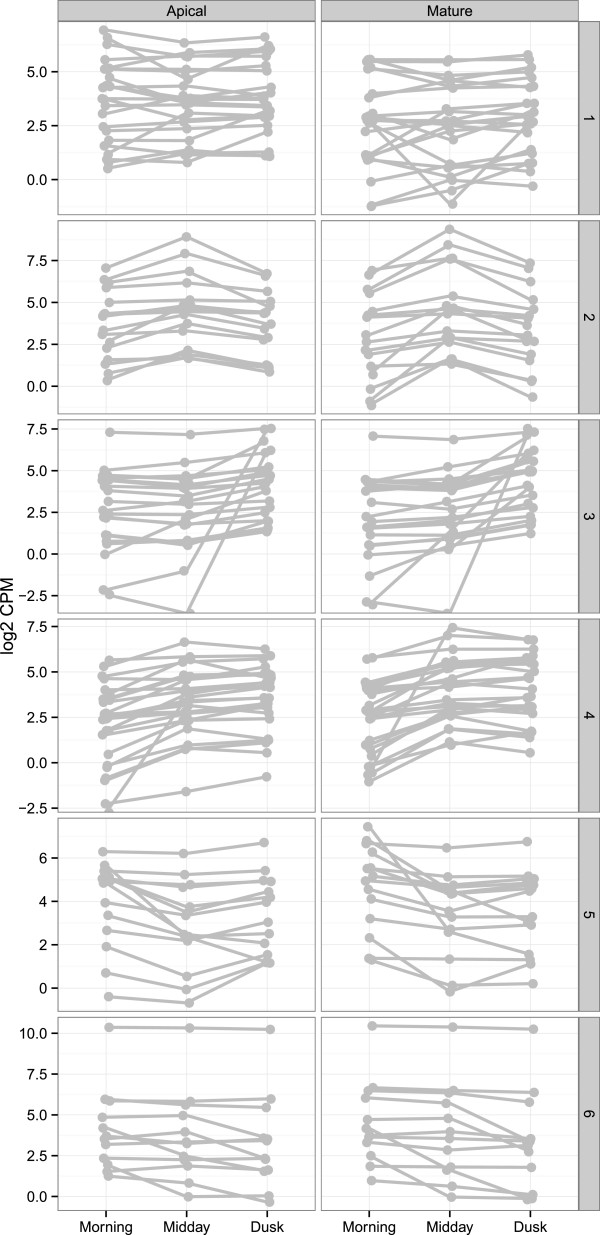
**Novel *R. stricta *genes, divided into clusters based on expression profile.** Novel proteins were identified from results of OrthoMCL analysis. The proteins were clustered based on expression profile using hierarchical clustering.

For comparison of orthologous protein families with other Eudicots we identified protein sequences based on BLAST results [50]. We used OrthoMCL [51] to find orthologous groups of proteins between other Eudicot species. OrthoMCL clusters protein families by first identifying within-species paralogs, based on BLAST score, and then identifies between species orthologus proteins by weighting for within-species paralog similarities. For comparison we used Arabidopsis and two genome sequenced Asterid species, tomato and potato (*Solanum lycopersicum* and *Solanum tuberosum*). Although these two species are closely related we reasoned that they represent a comprehensive resource of Asterid proteins and their combined usage would increase our confidence in identifying *Asteraceae*-specific proteins and *R. stricta* specific proteins. The results in Figure 8 show there are 360 protein families found in all three *Asteraceae* species and 880 protein families found uniquely in the *Solanaceae* species. However the majority of protein families (5596) are common to all species and large majority of protein families are shared between species. But the results do show that there are 293 protein families (628 proteins) unique to *R. stricta*, which is similar to that of the other species (tomato ~ 187, potato ~ 448 and Arabidopsis ~ 447). We then used the *R. stricta* specific protein families to identify novel genes which may be attributable to its adaptation. Of the 628 proteins, we extracted only those with an assigned GO function, since the ‘unknowns’ offered little further information. This reduced the number of proteins to 124. Hierarchical clustering was then used to identify proteins which were differentially expressed, from this we divided the data into six clusters, shown in Figure 9. Cluster 2 is associated with genes which are over-expressed at midday in both leaf types. We chose this expression pattern as it suggests that these genes may be involved in response to the increased temperature, VPD and light intensity at this time of the day. In this cluster we identified genes coding for 16 proteins which are shown in Table 5. These include a RAD23 homolog (R.stricta.12329 homologous to AT1G79650). In Arabidopsis RAD23 directs ubiquitinated proteins to the 26S proteasome for degradation [52]. Mutants defective in RAD23 have lethal phenotypes and this protein is involved in cell division [52]. Also a gene coding for a geranylgeranylated protein ATGP4 (R.stricta.17648 homologous to AT4G24990) was identified. Three genes encoding protein kinases (R.stricta.7380, R.stricta.23118, R.stricta.23205) and a ubiquitin protein gene (R.stricta.17648) were also identified. Additionally a homolog to AT3G08610 (R.stricta.8360), which codes for part of the Mitochondrial Respiratory Complex [53] was identified which might, along with R.stricta.25975, a gene coding for a component of respiratory chain electron transfer, may suggest that *R. stricta* has specific adaptations to mitochondrial respiration.

**Table 5:** Table of R. stricta unique genes over expressed at midday in both leaf types

**R.stricta ID**	**Description**	**ATG/Uniprot ID**
R.stricta.8360	Unknown protein	AT3G08610
R.stricta.8478	Putative uncharacterized protein	A5CBM0
R.stricta.12676	Homolog of mammalian P58IPK	AT5G03160
R.stricta.7380	Protein phosphatase 2C family protein	AT4G33500
R.stricta.10249	Tetratricopeptide repeat (TPR)-like superfamily protein	AT4G20900
R.stricta.12329	Rad23 UV excision repair protein family	AT1G79650
R.stricta.16783	Syntaxin/t-SNARE family protein	AT5G46860
R.stricta.17282	Unknown protein	AT2G35260
R.stricta.17648	Ubiquitin family protein	AT4G24990
R.stricta.21034	Uncharacterized protein	K4CDR0
R.stricta.21141	Type I (26 kD) CP29 polypeptide	Q00321
R.stricta.21402	Histone h1/h5, putative	B9SZV0
R.stricta.23118	MAP kinase 20	AT2G42880
R.stricta.23205	Cysteine-rich RLK (RECEPTOR-like protein kinase) 8	AT4G23160
R.stricta.25975	NADH:ubiquinone oxidoreductase subunit-17.2	Q8RXY1
R.stricta.26510	Uncharacterized protein	K4CFJ9

The table shows the R. stricta ID and description, with the corresponding homolog ID in either Arabidopsis or Uniprot.

## Discussion

Here we present the transcriptome profile of *R. stricta* quantified by RNA-seq. The transcriptome reconstruction produced 28018 transcripts (Figure 2b) which is similar to the number of genes found in other diploid plant species. For example, *Cucumis sativus* has ~28,000 genes [54] C*annabis sativa* ~30,000 [55] and Arabidopsis ~31,000 [56]. The average contig length (1643 bp, Figure 2b) suggests the vast majority are full length transcripts since the corresponding values of 1107, 1046 and 1551 bases for *C.sativus*, *C.sativa* and Arabidopsis respectively are of a similar size [54]–[56]. In substantiation of the integrity of this assembly, annotation assigned a function to 80% of the contigs (Figure 2c). Also a strong overlap with the sequenced genomes of other Eudicot species was found (Figure 8).

The BCV was found to be 19% within samples and 29% between sampling factors (Table 1). Typically, laboratory based RNA-seq experiments on Arabidopsis report BCVs of ~26% [57]. In general, we interpret this degree of variation in the data, comparable to plants in controlled environments, as reflecting the stability of the environment that *R. stricta* inhabits for long periods of time. The largest source of variation was the ‘midday’ samples (Table 1). At the outset of our field campaign in September 2011 it was known that VPD, temperature and humidity changed rapidly and reached a maximum 3 hours after dawn and stayed at this plateau for at least 5 hours (Figure 1e-f). This view on the predictability of *R. stricta’s* environment was reinforced by the same gene expression profiles being observed 12 months later in 2012 (Figure 4). This explains why the multivariate statistics was able to clearly assign RNA-seq data to specific sampling times and development state (Figure 3).

We found significant overlap between Arabidopsis circadian clock- regulated genes and their DE homologs in *R. stricta* in every pairwise time comparison (Table 2). In Arabidopsis, 30% of expressed genes are circadian regulated [26,58]. In *R. stricta*, 29-34% of all DE genes appear circadian regulated based on homologies to Arabidopsis genes regulated by the clock (Table 2). We are not in a position with *R. stricta* to test if the many homologs to Arabidopsis circadian-regulated genes are subject to this and/or a diel-regulated cue. However, in support of the argument here, we identified homologs of classic clock regulatory genes coding for TOC1, CCA1 and PRRs which showed an expression profile (Table 3) consistent with their Arabidopsis counterparts [42,43]. Moreover, many of the MYB and BHLH TF genes that were significantly differentially expressed in *R. stricta* (Figure 7) over a diel are implicated in circadian regulation in Arabidopsis [59].

As with other species [26,60,61], there is a clear adaptive advantage to *R. stricta* in placing many genes under circadian control. In the predictable environment *R. stricta* grows in, circadian- or diel-regulated processes can govern the timing of the appearance of expression of large groups of genes, while still remaining potentially responsive to unexpected environmental cues that could occur. This can allow anticipation of regular if extreme changes in the environment by the clock, modulating gene expression and restricting inappropriate responses, thus conferring a fitness benefit [61]–[63]. This reconfiguration of stress-responsive gene expression in advance of changes in the environment may be an adaptive feature of all plant species, even when growing in controlled environments [58,63,64]. For example, transcriptome profiling of drought-stressed Arabidopsis plants revealed only 10% of genes were differentially expressed as a consequence of stress imposition, the rest were circadian regulated [58].

We found increasing expression of NCED and ZEP genes (Table 3) which may drive changes in ABA content during the day consistent with the observed patterns of stomatal closure (Figure 1a; [24,27,65]). In addition in Arabidopsis, foliar ABA levels and signaling influence responses to high light as well as relative humidity [66,67]. Also we have seen no evidence for the significant differential expression of drought-responsive genes. Therefore it is unlikely that changes in the expression of NCED and ZEP genes (Table 3) reflect a response to severe water deficit. ABA levels have been shown to be regulated by the circadian clock, as is the expression of ABA-responsive genes [26] suggesting that the expression of genes coding for ABA metabolism and action in *R. stricta* is part of a daily cycle and not a stress response. Interestingly, the genes coding for enzymes involved in cytokinin and ethylene metabolism in Arabidopsis are not circadian regulated [26,62,68] and yet show very clear patterns of differential expression over the diel (Table 3). However again, from studies in Arabidopsis, the expression of these genes in *R. stricta* could be linked to changes in ABA metabolism. For example, cytokinin action has been shown to be mutually regulated with ABA in response to stress [69]. Conversely, the expression of genes of ethylene synthesis enzymes is the inverse of those involved in ABA biosynthesis [70]. The activity of salicylic acid signaling can negatively correlate with ABA signaling in Arabidopsis when subject to pathogen infection [64,70,71]. However in *R. stricta*, the expression of ICS genes increased throughout the day and peaked at dusk (Table 3). While classically associated with signaling to control resistance to biotrophic pathogens, salicylic acid is also important in eliciting thermotolerance [72] and decreasing susceptibility to photoinhibition induced by high light [73]–[75], a role for salicylic acid of perhaps greater relevance in *R. stricta*.

The above considerations comparing *R. stricta* with Arabidopsis prompt the question ‘do the daily changes in the transcriptome of *R. stricta*, reflect any stress response?’. Stress is defined as environmental conditions that reduce growth and yield below optimum levels [76]. It can be assumed that *R. stricta* is well adapted to its environment and the conditions it is normally exposed to, while extreme (Figure 1e-f), are a daily occurrence that fall within its tolerance range. Therefore, the large changes in the expression of many genes that were observed here are most likely not an exceptional response to stress, but part of the daily pattern for this plant. In summary, the large repertoire of DE genes over the day, annotated as stress-responsive or stress-defensive and which in temperate crops would indicate detrimental conditions, are for this species part of its routine diurnal cycle.

Despite the apparent predominance of circadian control of the *R. stricta* transcriptome, 66-71% of DE genes involved in responses to heat stress and salinity stress do not have circadian regulated homologs in Arabidopsis (Table 2). This could mean again that *R. stricta* differs from Arabidopsis and has brought these genes under circadian control. However, it may equally not be advantageous to have these genes regulated in this way. Heat stress responses may not be dictated entirely by the external environment but also by the internal leaf environment. The closure and opening of stomata in *R. stricta* may occur more frequently throughout the day [24]. Consequently, leaf internal temperature would fluctuate caused by changing evapo-transpiration. Furthermore, accompanying restrictions in photosynthesis leading to temporary increases in reactive oxygen species [77] may trigger changes in the expression of genes usually associated with heat stress. Heat shock protein and heat shock transcription factor gene expression is induced by ROS such as hydrogen peroxide (H_2_O_2_) in Arabidopsis and other species [78]–[80] and this may be the case in *R. stricta*. The potentially variable interactions between leaf internal temperature and ROS content may make it particularly important that such genes do not come under circadian control in this species.

Leaves of *R. stricta* have genes coding for components of non-photochemical quenching (NPQ) such as the enzymes of the xanthophyll cycle [81] (Additional file 5) and a homolog of PsbS (NPQ4; [82]). However, NPQ alone does not provide complete capacity to dissipate excess excitation energy under the types of high light conditions encountered by *R. stricta. R. stricta* photosynthesis is very resistant to photoinhibition, even at peak PPFDs [24]. To dissipate excess excitation energy under these conditions requires photochemical quenching processes [83]. The most prominent of these processes in *R. stricta* is photorespiration [24] and in support of this, the expression of many of the genes encoding enzymes of the photorespiratory cycle can be recognised (Additional file 5). Allied to this are a set of genes coding for enzymes of antioxidant and ROS metabolism (Additional file 5) and other enzymes and pathways that may play roles in photochemical quenching, such as the malate:oxaloacetate cycle (the malate valve; [84]) and PTOX. Most of the genes discussed here were not differentially expressed (the exception being PTOX and ZEP) and there is no means of assessing whether expression of the protective mechanisms in *R. stricta* are more highly expressed than in other plant species. Nevertheless, the clear presence of all these protective processes underscores their importance to *R. stricta*.

The identification of salinity-induced gene homologs in the DE gene set (Table 2) may indicate that at the Bahrah site, the plants were tapping groundwater with a high mineral content like *P. euphratica*[18]. However, groundwater in wadis is susceptible to changes in flow rate as a consequence of localized rainfall elsewhere in their catchment [12]. Therefore, changes in the mineral content of the groundwater supply to *R. stricta* may be variable, requiring a more flexible response independent of circadian regulation. This argument suggests that *R. stricta* experiences conditions in its natural environment reminiscent of those faced by irrigated crop plants. Irrigation does expose plants to water of varying mineral content and responses to salinity are important for crops in this type of agriculture [85]. The group of salinity-responsive genes identified in *R. stricta* may be worthy of more study in this context.

A considerable number of the DE genes in *R. stricta* may reflect more specialised adaptations to its desert environment. Highly significant overlaps were detected of *R. stricta* DE genes with smaller sized datasets from *P. euphratica* and *C. plantigineum* (Table 4; [17,18]). Overlapping of all datasets revealed a set of genes common to these three distantly related species (Table 4), which may reflect common challenges posed by their arid habitats. In particular DE genes coding for senescence-associated multiple cysteine proteinase isoforms [86] were detected in all three species. However, their expression is also responsive to a range of environmental stresses in other species [45]. Cysteine proteases are most likely to be involved in degradation of misfolded proteins, to maintain protein turnover under stress conditions [45]. Also in common were altered expression of RuBisCo activase (see also [24]) and raffinose synthesis genes (Table 4; see Results), which have both been found to have protective properties for high temperatures [45]–[47].

We identified a group of *R. stricta*-specific protein families (Figure 8) and then used the expression profiles of their genes to identify those that were over-expressed in the middle of the day (Figure 9). The rationale was to identify genes responsive to the increased temperature, light intensities and VPD at midday. Although we focused on this group, the other *R. stricta* specific-protein families without annotation detail and whose genes were not differentially expressed, may also include among them genes which are important for the adaptation of *R.stricta* to its environment. This is because these genes must have diversified and duplicated to give rise to a protein family, suggesting an evolutionary advantage. But given the large number of genes identified in this way, it is difficult to speculate on their function and such work is beyond the scope of this research. However of those genes that were differentially expressed, we did identify genes associated with photosynthesis and respiration. Work here and by Lawson *et al.,*[24] has highlighted the resilience of this species’ photosynthesis in this harsh environment. Complementary to this, our analysis may also indicate that modifications to respiration may be part of *R.stricta*’s adaptation of its primary metabolism. While there are several other genes worthy of comment (see Results), one very interesting gene is that encoding RAD23.The involvement of Arabidopsis RAD23 in the cell cycle [52] perhaps suggests that this more diversified protein family in *R. stricta* could be linked to a broader adaptive trait. In particular we see a significant enrichment of developmental GO terms (Figure 5) from the midday onwards. Therefore the apparent modulation of cell cycle and developmental genes in mature leaves could be part of a novel adaptive mechanism in *R. stricta*. However the work reported here is a transcriptomics study of a species in its natural environment and which, to our knowledge, has not been grown in the laboratory. Therefore, this and other potential novel adaptations in *R. stricta* await further technical developments to confirm the bioinformatic analyses reported here.

## Conclusions

The development of crop genotypes able to withstand greater climate change will require new sources of genetic variation. One source of such variation could be novel alleles in plant species adapted to more extreme environments. *R. stricta* is a perennial species dominant in its hot arid environment and yet displays a photosynthetic physiology typical of many major crop species [24]. As with all orphan species, identifying genes from *R. stricta* important for adaptation to its environment poses challenges in annotating novel genes and alleles. Moreover, recent studies on Arabidopsis has shown that determining the temporal pattern of stress responses is critical for distinguishing between genes subject to circadian or diurnal regulation and those responsive to the imposed stress [58]. Therefore, we determined the expression, over a diel, of the *R. stricta* foliar transcriptome *in situ* from field samples. We used these comprehensive datasets to evaluate the significance of stress-responsive mechanisms for the adaptation of this species. From early morning to dusk, changes in the transcriptome of *R. stricta* were extensive, 55% of 23000 quantifiable genes being differentially expressed. The results were highly reproducible with selected genes showing the same expression patterns 12 months later. These data highlight the potential of RNA-seq to investigate orphan plant species *in situ* with high precision. We found significant (P < 0.01) overlap between all pairwise comparisons of DE genes in *R. stricta* and Arabidopsis circadian regulated genes [26]. The many genes annotated as stress-responsive from laboratory studies are, in *R. stricta*, most likely to be subject to circadian regulation and represent a major adaptation to its predictable, if harsh, environment. Of the genes classified as not subject to circadian regulation, significant overlap were found of their expression profiles with genes from Arabidopsis responsive to salinity or combined heat and high light stress. This may reflect more unpredictable changes in the mineral content of groundwater and internal leaf temperature fluctuations. A comparison with more limited datasets from other arid zone species has identified several groups of genes commonly prominent to them all. Finally, we have identified genes which may represent adaptations not hitherto associated with improved tolerance to abiotic stress. Thus our comparative analyses and extensive temporal RNA-seq datasets have identified groups of genes for future study by the plant science community. Moreover this study reinforces that temporal profiling of global gene expression patterns is essential to identify potential stress-adaptive processes in plants.

## Methods

### Leaf material and sampling

All material was collected from a site at N21°26.456’, E39°31.847’ at Bahrah near Jeddah in the Kingdom of Saudi Arabia. Leaves were picked and snap frozen in liquid nitrogen and stored at -80°C until required for extraction. Apical leaves were those originating from the top crown of each branch, while mature leaves were picked from the fourth node down from the crown (Additional file 1). From each bush four apical and mature leaves were collected, one from each branch, i.e. for analysis four replicates were used. The two types of leaves were harvested from seven individual plants at the following times; A, 07:10; E, 12:40; F 13:25; G, 14:05; H, 14:30; L, 18:27 and Q, 10:40. Samples A and L were hereafter termed “morning” and “dusk” respectively. Sample A was collected immediately after sunrise and samples L collected immediately before sunset. For brevity, samples E-H and Q were designated collectively as “midday” samples. Samples A-L were collected on 18/10/2011 and Q 20/11/2011. For plants E and Q only apical leaves and mature leaves respectively were available for analysis.

### Leaf gas exchange and photosynthesis

All methods were performed exactly as described by Lawson *et al*[24].

### RNA extraction

Leaf tissue was ground to a fine powder in liquid nitrogen using a pestle and mortar, then *ca*. 100 mg of tissue was RNA extracted using Trizol^®^ (Life technologies, Carlsbad, USA) as described by [87]. The RNA was then purified using a Spin Column Reaction Cleanup Kit (NBS Biologicals, Huntingdon, UK) as described by the manufacturer. The amount of all samples were determined and their quality checked by electrophoresis through Tris-borate agarose gels (1% v/v; [88]) and by microfluidics electrophoresis using an Agilent (Agilent technologies, Santa Clara, USA) Bioanalyzer with RNA 6000 Nano Kit according to the manufacturer’s instructions. All RNA samples were stored at -80˚C.

### *RNA-seq* sequencing

RNA samples were sequenced by Genome Enterprise Limited (GEL) at The Genome Analysis Centre (Norwich, UK) across 8 lanes of an Illumina HiSeq 2000 (Illumina, San Diego USA); using 51 bp paired end reads, insert length ~200 bp. The data was de-multiplexed and quality checked using FastQC (http://www.bioinformatics.babraham.ac.uk/projects/fastqc/) by GEL. In total 1.72 billion paired end reads were returned for analysis which were uploaded to short read archive (SRA) as study SRP028238.

### Library normalisation

A multi-isolate sample was created using the total RNA samples above and other plant material, collected at the Barah site and a second site. This pool also comprised both apical and mature leaves. From the pooled sample 16 μg RNA was sent to Evrogen Ltd (Moscow, Russia) for library normalisation using service CS010-1A (http://www.evrogen.com/services/cDNA-normalization/service-cdna-normalization_Terms2.shtml) Briefly, total RNA sample was used for double-stranded cDNA synthesis using the SMART approach [89]. SMART-prepared amplified cDNA was then normalized using the duplex-specific nuclease (DSN) normalization method [90]. Normalisation included cDNA denaturation/re-association treatment (DSN, [91]) and amplification of the normalized fraction by PCR.

### 454 sequencing and quality checking

The normalised library prepared by Evrogen was sent to the Centre for Genomic Research (University of Liverpool, UK) for sequencing using 454 GS FLX platform (Roche, Basel, Switzerland). Data were quality checked using FastQC, and quality trimmed using clean_reads (http://bioinf.comav.upv.es/clean_reads) and all default settings. All bacterial reads were removed by aligning against all bacterial genomes downloaded from NCBI (ftp://ftp.ncbi.nlm.nih.gov/genomes/Bacteria/). Finally the SMART vector was removed using fastx_clipper (http://hannonlab.cshl.edu/fastx_toolkit/index.html) all sequences uploaded to SRA as study SRP028239.

### *De novo* transcriptome reconstruction

A flow diagram of the assembly process is shown in Figure 2. Briefly, the following steps were carried out:

### 454 assembly

Three assemblers were used for transcriptome SRP028239 reconstruction; CAP3 (version Date 10/15/07 [92]), MIRA (version 3.4.0.1, [93]) and GS De Novo Assembler (version 2.6, Roche Basel, Switzerland) (all default). To merge all three assemblers, CAP3 was used with increased stringency -o 40 and -p 90, to create the 454 super assembly.

### Illumina assembly

The SRP028238 data were divided into twelve pools (per plant and leaf type), then assembled first using Velvet (version 1.2.0.7 [94]) at two K-mers 37 and 43. Then Oases (version 0.2.0.8 [95]), was applied to the output from Velvet (both K-mers). Using the output from Oases, a Perl script was designed to select the ‘longest’ transcript when multiple isoforms are produced per locus (see Oases; [91]). The ‘longest’ contigs from the 37 and 43 k-mer assemblies were then combined using CAP3 (default settings). To merge the twelve individual assemblies a clustering strategy was used where all twelve assemblies were concatenated into a single file. Once concatenated CD-HIT-EST (version 4.5.4 [96]) was used with the following parameters: aS 0.4 -s 0.7 -aL 0.5. Clusters which had at least three contigs were selected using an in-house Perl script, to create the Illumina super assembly.

### Hybrid assembly

The 454 super assembly and Illumina super assembly were concatenated then clustered using CD-HIT-EST default settings (90% similarity). The representative cluster contigs were then split back into the assemblies which they were derived from (454 or Illumina), using an in-house Perl script. The Illumina assembly was then used to make a BLAST [97] database, to which the 454 contigs were searched using BLAST to find alignments with a minimum E-value 1e^-20^. 454 clusters without significant BLAST hits were then extracted and concatenated with the Illumina clusters to produce the final transcriptome.

### Annotation

For annotation of the transcriptome the sequences were matched by BLASTX against the UNIPROT (http://www.uniprot.org) database. These were then annotated for functional descriptions and GO terms. Contigs without functional description (or un-annotated) were then screened by BLASTX against canonical *Arabidopsis thaliana* (Arabidopsis) proteins from TAIR 10 [56] and annotated using TAIR 10 functional descriptions and GO terms. In all cases, BLASTX minimum e-value 1e^-15^ was used.

### Read mapping and transcript quantification

Bowtie (1, version 4.1.2 [98]) was used for read aligning; the reads were mapped using --best and --strata reporting, maximum 100 mappings per read and maximum insert length 1000 bp. The output Sam file was then directly used to estimate contig expression. This was done using an in-house Perl script and combination of UNIX commands to parse the data, and is freely available upon request. This uses the multi-fraction counting method as described by [99].

### Differential expression

To detect differentially expressed genes, EdgeR was used [57], the multi-fraction count data were rounded up to whole integers. For the analysis the following GLM model matrix was used (~Leaf + Time:Leaf), the nested model accounts for the unbalanced design of the experiment, where four plants were sampled at midday compared to just one at morning and dusk; by taking into account the effect of each leaf within the sampling time. As part of this method, transcripts with a count per million (CPM) expression profile of >2 CPM in at least four samples were retained. The biological coefficient of variation (BCV) was calculated from the square root of the common dispersion as described by [57].

### Gene ontology analysis

For gene ontology (GO) analysis TopGO was used from Bioconductor in R [100]. This was done for all DE genes, split between over and under-expressed genes identified by EdgeR, for each pairwise comparison. Fishers test was implemented in TopGO to identify enriched GO terms per comparison. From this, all GO terms with a *P* < 0.01 were selected. Then Cytoscape [101] was used to visualise the selected GO terms, using Arabidopsis GO network.

### Mapman

To use Mapman [102], a mapping file was created using the BLAST results described above. This was used to find homologus TAIR identifiers and then parse the corresponding Bin codes from Mapmans Arabidopsis mapping file and convert to *R. stricta*. For experimental data the log(2)FC values calculated from EdgeR were used. All files are available upon request.

### Arabidopsis microarray data comparisons

For comparisons with microarray data in Arabidopsis, the NCBI series GSE41935 was used and the robust multi-array average (RMA; [103]) values were downloaded. The dataset includes accession Col-0 subjected to a series of single and double abiotic stress experiments [25]. For calculation of DE genes one-way ANOVA was applied for treated *vs.* control. Homologs between Arabidopsis and *R. stricta* were identified by BLASTX of *R. stricta* (nucleotide) against Arabidopsis canonical proteins (filter e < 1e^-15^). For enrichment test, hypergeometric tests (phyper) were used in R, the results were Bonferroni-corrected (90 tests in total). For circadian genes, a meta-analysis [26] (the C + E intersection group) was used.

For comparison with *P. euphratica* data [18], all ESTs which were differentially expressed were downloaded from Genbank, then screened using BLASTX against TAIR proteins to find homologs. These were then compared in the same way as the Arabidopsis microarray datasets to find overlap. Comparison with *C. plantagineum* was not possible using homology based comparison as the sequences used by [17] were not available. Instead these comparisons were made using the functional and GO terms described by [17].

### Quantitative PCR

cDNA was made using First Strand cDNA synthesis (Thermo-scientific, Waltham, USA) as described by the manufacturer, using random hexamers for priming. Quantitative (Q)-PCR reactions were made using Bioline (London, UK) sensiFAST SYBR master mix and performed on a Bio-Rad CFX-96 (Bio-Rad Laboratories, Hercules, USA) as described by the manufacturer. The relative expression software tool (REST-384) was used to calculate the relative fold change in gene expression (http://www.gene-quantification.info/) [104] using the mathematical model described by Pfaffl [105]. Primer efficiencies were measured using a serial dilution of stock cDNA at 1:1, 1:10 and 1:100. All primers are shown in Additional file 6. R.stricta.10855, ‘Chromatin remodeling complex subunit’ was chosen as the reference gene, this was chosen as it had the lowest variation and stable expression across all samples (mean CPM 5.45 and standard deviation 0.12). Each treatment was replicated with three biological samples, and each biological sample included three technical replicates. In addition, cDNA was also made and QPCR tested from mature leaves collected in September 2012 at the same time periods and location. For all Q-PCR estimates the fold change in gene expression was quantified using the ‘morning’ sample was used as the ‘control’. All calculations were made for each leaf type and year.

### Orthologous protein families

To identify protein coding sequences in *R. stricta* the transcriptome was searched against the *A.thaliana* (TAIR10) protein database, above, with minimum e-value 1e-5. To identify open reading frames OrfPredictor was used [50] using the offline Linux version 3.0. All protein sequences are available upon request.

For comparison of orthologous genes, the derived protein sequences for *Solanum lycopersicum* and *Solanum tuberosum* were downloaded from Ensembl [106] using the Perl API tool [107]. To ensure that full length proteins were downloaded once for each gene, canonical proteins were downloaded [108]. Arabidopsis (TAIR10) protein sequences were also used for comparison. To identify orthologous protein sequences OrthoMCL [51] was used.

### Novel differentially expressed genes

To identify protein families unique to *R. stricta*, the unique protein family proteins identified from orthologous analysis (293 families ~ 623 proteins (Figure 8)) were extracted. The expression patterns were then subject to cluster analysis. The CPM data was first scaled (by subtracting mean across treatments) then Euclidean distance calculated and hierarchical clusters determined for six groups using the ‘Ward’ method using R, ‘hclust’ function.

### Statistical analysis and Graphs

All statistical analysis was done using R [109].

## Competing interests

The authors declare that they have no competing interests.

## Authors contributions

SAY carried out bioinformatics analysis, QPCR and drafted manuscript and IC assisted in script writing for bioinformatics. RAF designed and carried out RNA extraction protocol, UB participated in experimental design and draft of manuscript. MB and , MZM participated in the coordination and sampling of the experimental design. Both NB and JS conceived the study and assisted drafting of manuscript. TL performed and analysed all of the photosynthetic measurements. PMM conceived and coordinated the study and drafted the manuscript. All authors read and approved the final manuscript.

## Supplementary Material

Additional file 1 **Is a figure showing *Rhazya stricta *** **plants at sampling location and leaves.** Click here for file

Additional file 2 Is a table showing the transcriptome annotation details including GO terms. Click here for file

Additional file 3 Is a table showing the average CPM and results of test for significant differential expression of contigs. Click here for file

Additional file 4 **Is a table showing the overlap between *R. stricta *** **and Arabidopsis microarray data.** Click here for file

Additional file 5 Is a list of protective enzymes. Click here for file

Additional file 6 Is a table showing quantitative PCR primers. Click here for file
