# Supplementary material for: The temporal foliar transcriptome of the perennial C3 desert plant Rhazya stricta in its natural environment
Source: BMC Plant Biol. 2014 Jan 4;14:2. doi: 10.1186/1471-2229-14-2 (PMC3906910; doi:10.1186/1471-2229-14-2)

Supplmental Figure 1. *Rhazya stricta* plants at sampling location.

a) shows plants at the Barah site b) shows sampling for apical leaves

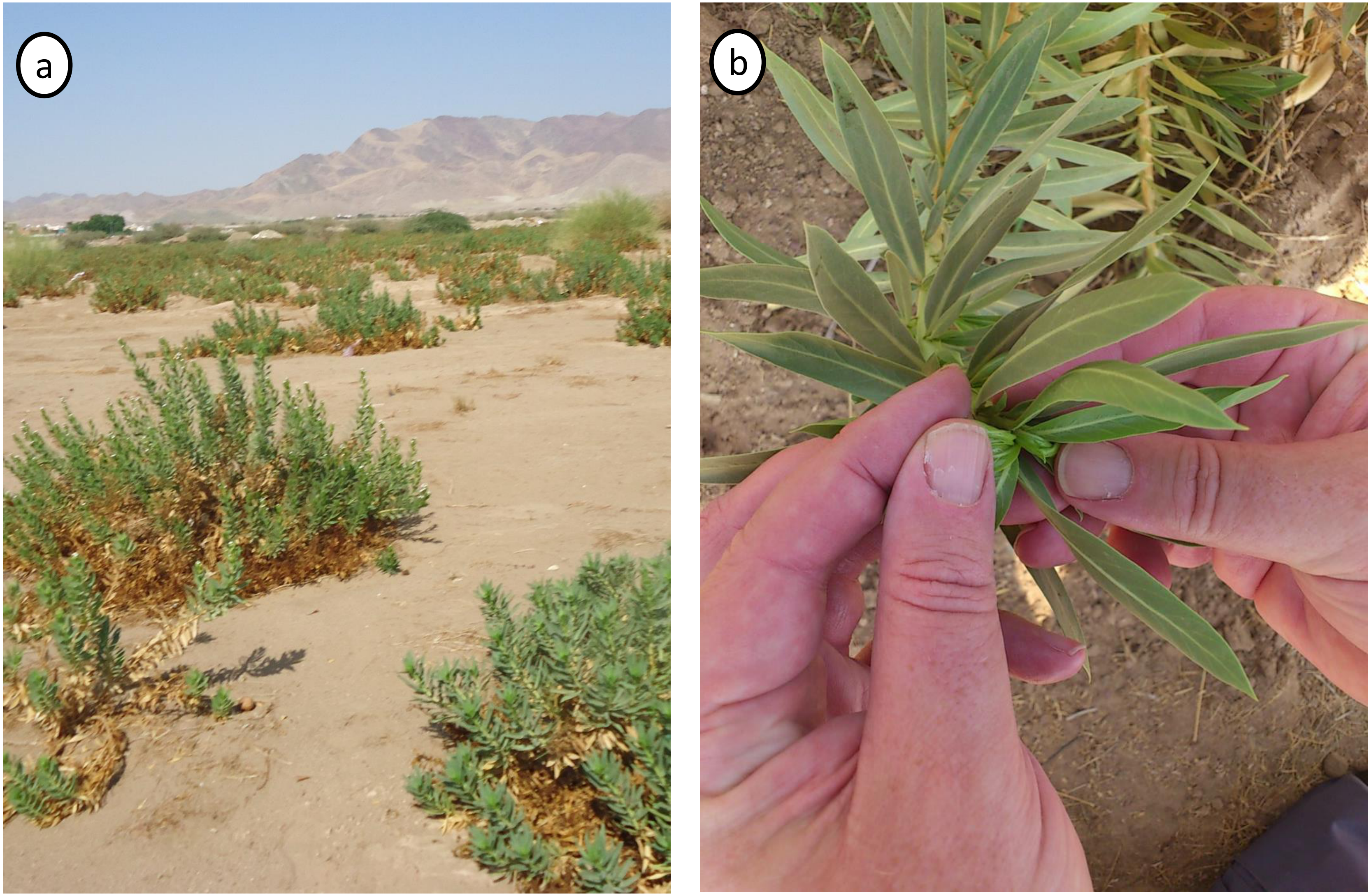

Supplement: Additional file 1 — Is a figure showing Rhazya stricta plants at sampling location and leaves. [file 1471-2229-14-2-S1.pdf]
