# Supplementary material for: The temporal foliar transcriptome of the perennial C3 desert plant Rhazya stricta in its natural environment
Source: BMC Plant Biol. 2014 Jan 4;14:2. doi: 10.1186/1471-2229-14-2 (PMC3906910; doi:10.1186/1471-2229-14-2)
Supplement: Additional file 5 — Is a list of protective enzymes. [file 1471-2229-14-2-S5.pdf]

Supplemental Table 5. Protective enzymes

ID	Process
R.stricta.18151	Photorespiration
R.stricta.21224	Photorespiration
R.stricta.25724	Photorespiration
R.stricta.4377	Photorespiration
R.stricta.5586	Photorespiration
R.stricta.7227	Photorespiration
R.stricta.6844	Photorespiration
R.stricta.9898	Photorespiration
R.stricta.17161	Photorespiration
R.stricta.4463	Photorespiration
R.stricta.4478	Photorespiration
R.stricta.12582	Photorespiration
R.stricta.21246	Photorespiration
R.stricta.20361	Photorespiration
R.stricta.21161	Photorespiration
R.stricta.23984	Photorespiration
R.stricta.14031	Photorespiration
R.stricta.6885	Photorespiration
R.stricta.19996	Photorespiration
R.stricta.23421	Photorespiration
R.stricta.24773	Photorespiration
R.stricta.1433	Photorespiration
R.stricta.2365	Photorespiration
R.stricta.10633	Xanthophyll cycle
R.stricta.12393	Xanthophyll cycle
R.stricta.13114	Xanthophyll cycle
R.stricta.15435	NPQ4
R.stricta.17543	PTOX
R.stricta.18857	PTOX
